# Five-year outcomes of stereotactic body radiation therapy (SBRT) for prostate cancer: the largest experience in China

**DOI:** 10.1007/s00432-021-03785-2

**Published:** 2021-09-15

**Authors:** Xianzhi Zhao, Yusheng Ye, Haiyan Yu, Lingong Jiang, Chao Cheng, Xueling Guo, Xiaoping Ju, Xiaofei Zhu, Huojun Zhang

**Affiliations:** 1 Department of Radiation Oncology, Shanghai Changhai Hospital, The Second Military Medical University, No. 168 Changhai Road, Shanghai, 200433 China; 2 Department of Oncology, Xuanhan County People’s Hospital, Dazhou, 636150 China; 3 Department of Nuclear Medicine, Shanghai Changhai Hospital, The Second Military Medical University, Shanghai, 200433 China

**Keywords:** Prostate cancer, Stereotactic body radiation therapy (SBRT), CyberKnife, Biochemical progression-free survival (bPFS), Toxicity

## Abstract

**Objective:**

To evaluate the efficacy and safety of SBRT for localized prostate cancer (PCa) with CyberKnife in China. Moreover, it is the largest-to-date pilot study to report 5-year outcomes of SBRT for localized PCa from China.

**Methods:**

In this retrospective study, 133 PCa patients in our center were treated by SBRT with CyberKnife (Accuray Inc., Sunnyvale, USA) from October 2012 to July 2019. Follow-up was performed every 3 months for efficacy and toxicity evaluation. Biochemical progression-free survival (bPFS) and toxicities were assessed using the Phoenix definition and the Common Terminology Criteria for Adverse Events (CTCAE) v.5.0, respectively. Factors predictive of bPFS were identified with COX regression analysis.

**Results:**

133 patients (10 low-, 21 favorable intermediate-, 31 unfavorable intermediate-, 45 high-, and 26 very high risk cases on the basis of NCCN risk classification) with a median age of 76 years (range 54–87 years) received SBRT. The median dose was 36.25 Gy (range 34–37.5 Gy) in 5 fractions. Median follow-up time was 57.7 months (3.5–97.2 months). The overall 5-year bPFS rate was 83.6% for all patients. The 5-year bPFS rate of patients with low-, favorable intermediate-, unfavorable intermediate-, high-, and very high risk PCa was 87.5%, 95.2%, 90.5%, 86.3%, and 61.6%, respectively. Urinary symptoms were all alleviated after SBRT. All patients tolerated SBRT with 1 (0.8%) patient reporting grade-3 acute and 1 (0.8%) patient reporting grade-3 late genitourinary (GU) toxicity, respectively. There were no grade 4 toxicities. Gleason score (*P* < 0.001, HR = 7.483, 95%CI: 2.686–20.846) was the independent predictor of bPFS rate after multivariate analysis.

**Conclusion:**

SBRT is an efficient and safe treatment modality for localized PCa with high 5-year bPFS rates and acceptable toxicities.

## Introduction

PCa is the most common cancer in men and the leading cause of death among malignancy entities (https://gco.iarc.fr/). For localized PCa, daily target location with image-guided radiotherapy (IGRT) is essential with intensity-modulated radiotherapy (IMRT) for target margin reduction and treatment accuracy, which has been recommended as one of the standard therapies (National Comprehensive Cancer Network: NCCN Clinical Practice Guidelines in Oncology: Prostate Cancer (Version 4.2019) 2019). Conventionally fractionated IMRT with 1.8–2.0 Gy per fraction has been used increasingly in practice. What’s more, radiotherapy combined with androgen deprivation therapy (ADT) is adopted for unfavorable intermediate, high and very high risk groups (National Comprehensive Cancer Network: NCCN Clinical Practice Guidelines in Oncology: Prostate Cancer (Version 4.2019) 2019).

PCa has a unique radiobiological feature, namely the relatively slow proliferation, characterized by a low *α*/*β* ratio compared to the normal organs around the target (Brenner and Hall 1999; Fowler et al. 2003). The *α*/*β* ratio of PCa is about 1.5 Gy, while that of the rectum and bladder is about 3.0 Gy. Owing to the characteristic, extreme hypofractionated radiotherapy would offer favourable tumor control without increasing risk of late toxicity.

Recently, due to the advantages of SBRT with highly conformal and precise image-guided delivery, growing evidence has confirmed its pivotal role in tumor control. Moreover, it has been commonly used in localized PCa patients, showing excellent bPFS rates and tolerable toxicities, especially when surgery is unsuitable or declined (Yu et al. 2014; Freeman and King 2011; Kishan and King 2017). Majority of studies using SBRT indicated that the 5-year bPFS for patients with low-, intermediate-, and high-risk PCa was 95%, 84% and 81%, respectively (King et al. 2013). However, all these studies were performed in Caucasians. Compared with Western patients, the genomic alteration signatures in Chinese cohorts were obviously different (Li et al. 2020). In fact, the clinical utility of SBRT for PCa in Chinese population has been rarely reported. Our study aims to assess the toxicity and efficacy of SBRT for localized PCa in Chinese population.

## Materials and methods

### Patient selection

All patients were retrospectively screened for eligibility by an oncologist before the study. The inclusion criteria included histologically confirmed adenocarcinoma of the prostate, at least imaging examinations with enhanced pelvic magnetic resonance imaging (MRI) and positron emission computed tomography (PET CT), an Eastern Cooperative Oncology Group (ECOG) score ≤ 1, no involvement of regional lymph nodes or distant metastasis. Patients who refused or were not suitable for surgery because of underlying diseases were enrolled for screening. The informed consent was obtained from all enrolled patients before the treatment. The study was performed based on the Declaration of Helsinki and was approved by our institutional review board.

### SBRT protocols

Before radiotherapy planning, fours gold fiducials were placed into the prostate. The patients were immobilized by thermoplastic body mask in supine position with arms by their sides. One week after fiducial placement, enhanced computed tomography (CT) scan was performed with a slice thickness of 1.5 mm, and the scan range of at least 10 cm below and above the prostate. In the meanwhile, MRI imaging was required for all patients. Fused MRI and CT images were then used for target and organs at risk (OARs) delineation. For low risk PCa cohorts, the clinical target volume (CTV) included the whole prostate. For favorable and unfavorable intermediate risk grouping, CTV included the whole prostate and 1 cm of the seminal vesicles whereas CTV included the whole prostate and 2 cm of the seminal vesicles for high and very high risk groups. SBRT was delivered by CyberKnife (Accuray Corporation, Sunnyvale, CA, USA). Planning target volume (PTV) was delineated with a 5 mm margin expansion in all directions except for posterior direction with a 3 mm expansion from CTV to decrease the excessive radiation of the rectum. The treatment parameters were presented in Table 1. The prescription doses of 34–37.5 Gy in 5 fractions were delivered to the PTV every other day with a median prescription isodose line of 79%. The dose-volume constraints for OARs were as follows: for the rectum, V18.1 Gy < 50%, V29 Gy < 20%, V36 Gy < 1 cc; for the bladder, V18.1 Gy < 40%, V37 Gy < 10 cc (optimal V37 Gy < 5 cc); for the prostatic urethra: V42 Gy < 50%; for the femoral head, V14.5 Gy < 5%; for the penile bulb, V29.5 Gy < 50%; for the bowel, V18.1 Gy < 5 cc, V30 Gy < 1 cc (Henderson et al. 2015).

**Table 1:** Treatment parameters used for SBRT

Parameters	All lesions
CTV (ml)	50.0 (10.0–182.7)
Maximum dose (Gy)	45.9 (42.7–58.6)
Total prescribed dose (Gy)	36.25 (34–37.5)
Number of fractions	5 (5)
Dose per fraction (Gy)	7.25 (6.8–7.5)
BED_1.5_ (Gy)	211.5 (188.1–225)
Number of fiducials	4 (2–5)
Prescription isodose line (%)	79 (65–85)

### Response evaluation and follow-up

The patients’ prostate-specific antigen (PSA) as well as the testosterone levels were checked every month. Biochemical progression was defined as PSA increased ≥ 2 ng/mL from nadir (Roach et al. 2006). Biochemical progression-free survival (bPFS) was defined as the time from the date of SBRT delivery to the biochemical progression or the last follow-up. Local control (LC) was defined as local prostate lesions without progression. Overall survival (OS) was defined as the time from the beginning of radiation therapy to the last follow-up or death. Disease progression free survival (DPFS) was defined as the time from the date of the beginning of radiation therapy to any sites with clinical tumor progressions or death. Acute and late toxicity was scored according to CTCAE v 5.0.

### Statistical analysis

Biochemical progression-free survival (bPFS) rates were calculated by the Kaplan–Meier method. Potential factors associated with bPFS were identified with univariate and then multivariate COX proportional hazards regression model. SPSS 18.0 (IBM Corporation, Armonk, NY, USA) was applied for statistical analyses. *P* value < 0.05 was considered as statistically significant.

## Results

### Patients’ characteristics

SBRT was delivered to 133 localized PCa patients (10 low-, 21 favorable intermediate-, 31 unfavorable intermediate-, 45 high-, and 26 very high risk cases according to the NCCN risk classification) with a median age of 76 years (range 54–87 years) from October 2012 to July 2019 in Shanghai Changhai Hospital of the Second Military Medical University. The median pre-treatment PSA was 12.05 ng/mL (range 0.03–104.8 ng/mL). Of all patients, 18 (13.5%) patients had two primary cancers and 1 (0.8%) patient had three. According to the NCCN Guideline (2021 V2), we suggested low and favorable intermediate risk group patients without ADT, unfavorable intermediate risk group patients with ADT 4–6 months, high and very high risk group patients with ADT 1.5–3 years. However, some patients refused to apply ADT in the study. There were 50 patients present of ADT. And the median time of ADT was 18.5 months (range 0.6–156.0 months). Their baseline characteristics were summarized in Table 2.

**Table 2:** Patient demography and clinical presentation

Characteristics	Values	Characteristics	Values
Age (years)	76 (range 54–87)	Pre-treatment hormone treatment	
Gleason score			
1 + 3	1 (0.8%)	Yes	36 (27.1%)
3 + 2	2 (1.5%)	No	97 (72.9%)
3 + 3	41 (30.8%)	Stage	
3 + 4	25 (18.8%)	T2a	43 (32.3%)
4 + 3	22 (16.5%)	T2b	9 (6.8%)
4 + 4	23 (17.3%)	T2c	49 (36.8%)
3 + 5	1 (0.8%)	T3a	1 (0.8%)
5 + 3	2 (1.5%)	T3b	6 (4.5%)
4 + 5	9 (6.8%)	T4	25 (18.8%)
5 + 4	6 (4.5%)	Tx	16 (12.0%)
5 + 5	1 (0.8%)	NCCN risk grouping	
Median (interquartile range) PSA at diagnosis (ng/ml)	15.98 (9.1–26.2)	Low	10 (7.5%)
Pre-treatment PSA (ng/ml)		Favorable intermediate	21 (15.8%)
< 10	52 (39.1%)	Unfavorable intermediate	31 (23.3%)
10–20	47 (35.3%)		
> 20	34 (25.6%)	High	45 (33.8%)
Symptoms			
Presented	59 (44.4%)	Very high	26 (19.5%)
None	74 (55.6%)	Pre-treatment TURP	
ECOG score		Yes	25 (18.8%)
0	5 (3.8%)	No	108 (81.2%)
1	128 (96.2%)		

### Outcomes

The follow-up for the cohort was until July 2020 or death. The median follow-up was 57.7 months (3.5–97.2 months). 10 patients (7.5%) were dead. Non cancer-specific death was found in 4 patients (2 patients with cerebral infarction, 1 with pneumonia and 1 with Parkinsonian syndrome), while 4 patients died of PCa metastasis and 2 patients died of progressions of other tumors. For the non-cancer-specific death of 4 patients, one was with 1 unfavorable intermediate-, one with high-, and two were with very high risk according to the NCCN risk classification); for the 4 patients died of PCa metastasis, one very high risk case died from abdominal metastasis, while the remaining 3 cases died from bone metastasis. The 2- and 5-year OS rates were 99.2% and 93.0%, respectively. The 2- and 5-year LC rates were 99.2% and 96.1%, respectively. The 2- and 5-year DPFS rates were 96.1% and 88.1%, respectively.

**Fig. 1:**
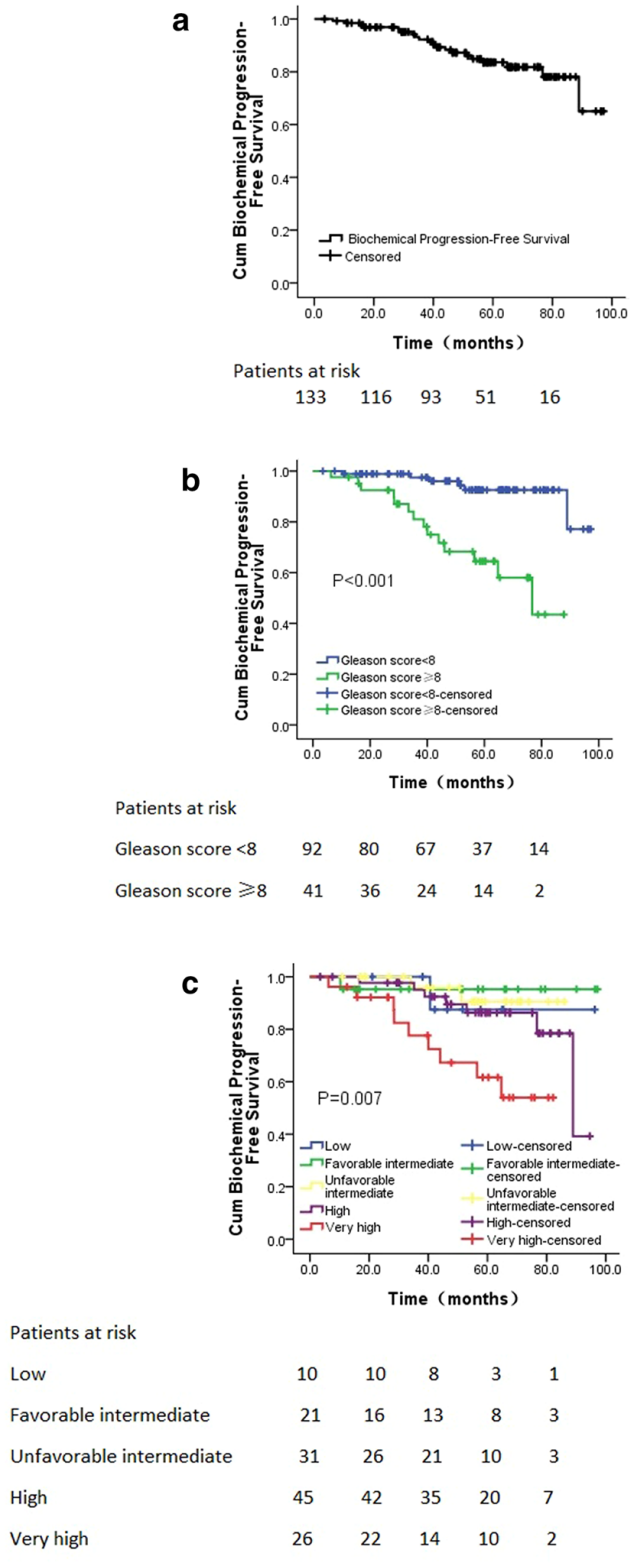
Actuarial survival analysis of patients. **a** Overall bPFS. **b** bPFS in different Gleason score. **c** bPFS in different NCCN risk groupings*. bPFS* biochemical progression-free survival*, Cum *cumulative

**Fig. 2:**
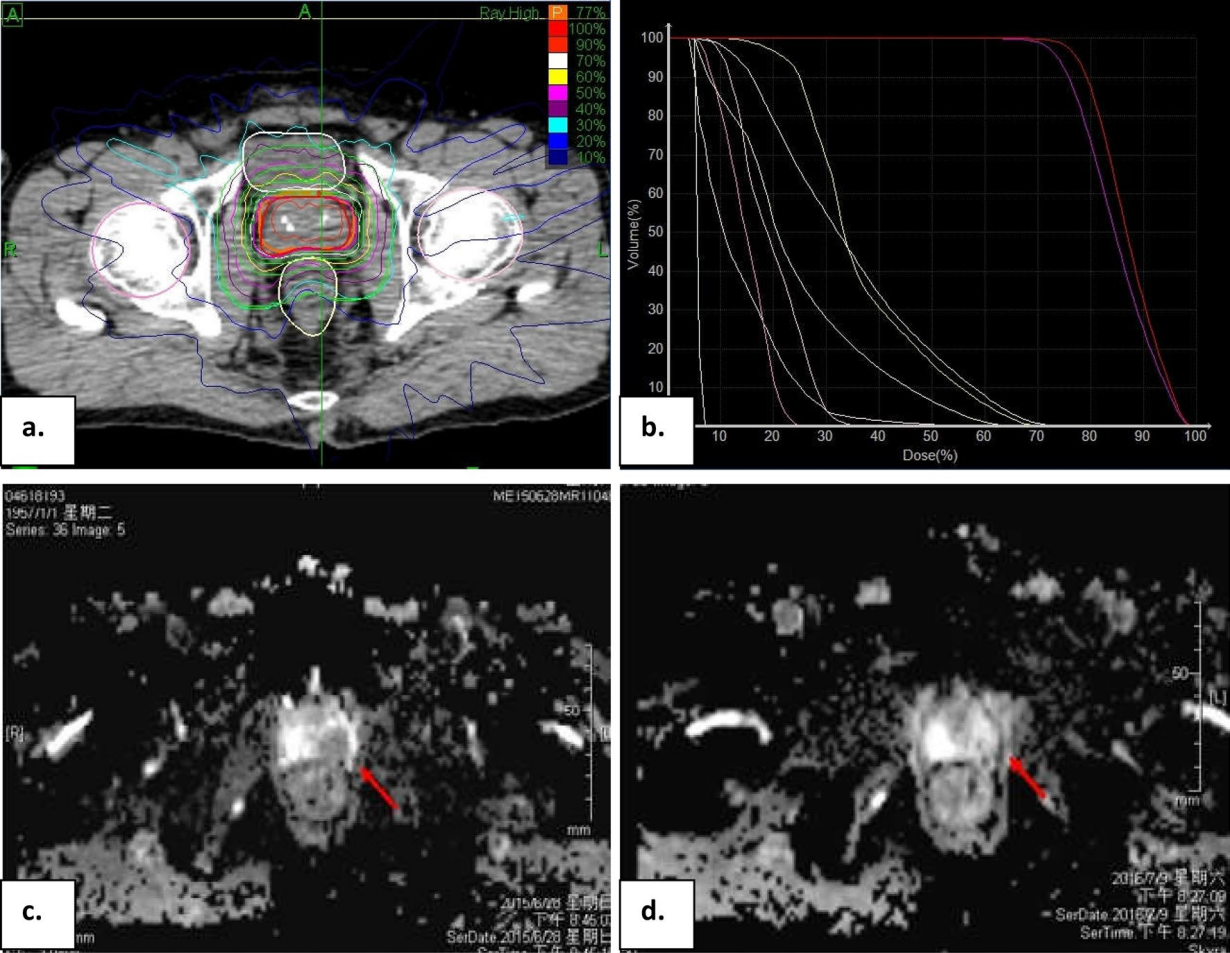
An illustrative case of successful SBRT for 58-year-old man with unfavorable intermediate risk prostate cancer. **a** CT scan before SBRT and 36.25 Gy in 5 fractions was prescribed for prostate cancer. **b** A typical DVH for Cyberknife treatment of a prostate cancer patient is shown, revealing doses to the CTV, PTV, and nearby critical structures. Take the abscissa as reference, the organizations and targets from left to right are as follows: penile bulb, right femoral head, left femoral head, bowel, urethra, rectum, bladder, PTV, CTV. **c** Enhanced MRI scan before SBRT. **d** Enhanced MRI scan 1 year after SBRT. *SBRT* stereotactic body radiation therapy, *CT* computed tomography, *DVH* dose-volume histogram, *CTV* clinical target volume, *PTV* planning tumor volume. The red arrows indicate tumor location before and after SBRT

Furthermore, the 2- and 5-year bPFS rates were 96.9% and 83.6%, respectively (Fig. 1a). In details, the 2 and 5-year bPFS rates for patients with low-, favorable intermediate-, unfavorable intermediate-, high-, and very high risk PCa were 100% and 87.5%, 95.2% and 95.2%, 100% and 90.5%, 100% and 86.3%, 96.2% and 61.6%, respectively (*P* = 0.007, Fig. 1c). In the univariate analysis, patients with Gleason score < 8 had a high bPFS rate than those with Gleason score ≥ 8 (*P* < 0.001, Fig. 1b). Moreover, patients without ADT had a high bPFS rate than those with ADT (*P* = 0.004, Table 3). However, only Gleason score (*P* < 0.001, RR = 7.483, 95%CI: 2.686–20.846) was the independent predictors of bPFS rate after multivariate analysis. No significant correlation was found between bPFS rate and CTV volume (*P* = 0.985), pre-treatment PSA level (*P* = 0.253), symptoms (*P* = 0.773) or age (*P* = 0.903). It was illustrated in Table 3. Additionally, an illustrative case was shown in Fig. 2.

**Table 3:** Univariate analysis for bPFS rate

Factors	2-year b-PFS rate (%)	5-year b-PFS rate (%)	*P* value
CTV (ml)			
< 50	95.4	85.3	0.985
≥ 50	98.5	82.2	
Gleason score			
< 8	98.9	92.5	** < 0.001**
≥ 8	92.5	64.5	
PSA at diagnosis (ng/ml)			
< 10	94.4	86.0	0.774
10–20	96.0	86.3	
> 20	100	80.2	
Pre-treatment PSA (ng/ml)			
< 10	94.0	88.4	0.253
10–20	97.8	87.1	
> 20	100	74.2	
Symptoms			
Presented	98.2	85.9	0.733
No	95.8	81.5	
NCCN risk grouping			
Low	100	87.5	
Favorable int ermediate	95.2	95.2	
Unfavorable intermediate	100	90.5	**0.007**
High	97.7	86.3	
Very high	92.1	61.6	
Age (years)			
< 70	93.4	80.0	0.903
≥ 70	98.0	84.7	
ADT			
Presence	95.8	73.2	**0.004**
Absence	97.6	90.4	

The bold vaues indicate statistical significance

The symptoms of urethral obstruction and irritative symptoms were commonly found in most patients, which included dysuria, frequency of micturition, nocturnal frequency of micturition, urodynia and urgency of urination. Fifty-nine patients complained of one or more urinary symptoms. All of them (100.0%) had alleviation of symptoms after radiotherapy.

### Treatment toxicity

SBRT was well-tolerated for the majority of patients. There was only one patient (0.8%) reporting grade 3 acute and late genitourinary (GU) toxicity with radiation cystitis. No grade 4 or higher adverse reaction was observed. Two (1.5%) patients had grade 2 acute GU toxicity, 1 (0.8%) with grade 2 acute gastrointestinal (GI) and 3 (2.3%) with grade 2 late GU toxicity. Hematuria, frequent urination, increased frequency of nocturia, painful urination and difficult urination were the most common adverse effects during treatment. All acute toxicities were transitory, it was reversed and improved by medication, which did not prevent patients from completing the treatment (Table 4).

**Table 4:** Acute and late urinary and rectal toxicity on the RTOG scale for prostate cancer patients after SBRT

Toxicities	Grade 1	Grade 2	Grade3	Grade4
Acute GU	10 (7.5%)	2 (1.5%)	1 (0.8%)	0 (–)
Acute GI	3 (2.3%)	1 (0.8%)	0 (–)	0 (–)
Late GU	5 (3.8%)	3 (2.3%)	1 (0.8%)	0 (–)
Late GI	0 (–)	0 (–)	0 (–)	0 (–)

*PCa* prostate cancer

## Discussion

The study investigated the efficacy and toxicity of SBRT for localized PCa. Overall, SBRT may offer a higher 5-year bPFS rate of 83.6% and effective symptom relief without severe adverse effects. Additionally, no grade 4 or above adverse reactions were reported. Thence, it may provide evidence that SBRT was also a promising treatment for localized PCa in Chinese population. To the best of our knowledge, this is the longest follow-up and largest study to report SBRT for localized PCa from China.

Theoretically, due to low *α*/*β* of the prostate cancer, a high single dose could improve tumor control and reduce the risk of late toxicity in bladder and rectum. Three large studies identified the average *α*/*β* ratio of PCa was less than 2 Gy: (1) Proust-Lima et al. (2011) analyzed 5093 patients with *α/β*  = 1.55 Gy (95% CI = 0.46–4.52); (2) Miralbell et al. (2012) analyzed 5969 patients with *α*/*β*  = 1.4 Gy (95%CI = 0.0.9–4.2); (3) ASTRO guideline evaluated 14,168 patients with *α/β* = 1.7 Gy (95%CI = 1.4–2.2) and *α/β* = 1.6 Gy (95%CI = 1.2–2.2) was applied by Phoenix criteria (Dasu and Toma-Dasu 2012; Dasu 2007). Although SBRT has been confirmed as an effective option, it still remains controversial whether SBRT has an advantage over conventional fractionated radiotherapy which is the current standard of care in terms of outcomes and toxicities. The two ongoing phase 3 clinical trials of HYPO trial and PACE study tried to provide answers. Intermediate to high risk PCa patients were recruited in the HYPO trial, in which 78 Gy in 39 fractions daily was compared with 42.7 Gy in 7 fractions given in every other day (ISRCTN 2014). The 5-year outcomes supported the use of SBRT for radiotherapy of PCa. On one hand, 5-year failure-free survival in SBRT group and conventional fractionation group were 84% (95% CI: 80–87) and 84% (95% CI: 80–87), respectively (*P* = 0.99). Hence, for intermediate-to-high risk PCa, the failure-free survival in SBRT group was non-inferior to conventionally fractionated radiotherapy group. On the other hand, no obvious differences between SBRT group (11 [5%] patients) and conventional fractionation group (12 [5%] patients) in frequencies at 5 years of RTOG grade 2 or higher GU adverse reaction (*P* = 1.00) was observed. And there was no difference in GI adverse reaction (3 [1%] patients vs 9 [4%] patients; *P* = 0·14) between these two groups. Late toxicities were similar in both groups whereas early adverse effects were more common with SBRT compared with conventional fractionation (Widmark et al. 2019). Regarding to the PACE study, low and intermediate risk PCa patients were enrolled (UKCRN 2014; Tree 2013). It included two trials: PACE-A and PACE-B. In PACE-A study, the suitable patients for prostatectomy were randomized into laparoscopic surgery and SBRT. In PACE-B study, patients were randomized into image-guided IMRT and SBRT. The prescription dose of IMRT was 78 Gy in 39 fractions, while the prescription dose of SBRT was 36.25 Gy in 5 fractions or 38 Gy in 4 fractions. The acute toxicity of PACE-B was reported in 2019. There was no significant statistical difference with grade 2 or more GI toxicity between conventionally fractionated radiotherapy group and SBRT group (12% [53/432] patients vs 10% [43/415] patients, *P* = 0.38), in the meanwhile, no significant differences in grade 2 or worse GU toxicity were observed in both groups (27% [118/432] patients vs 23% [96/415] patients, *P* = 0.16). The results suggested that substantially shortening treatment courses with SBRT didn't increase either acute GI or GU toxicity (Brand et al. 2019).

Regarding to a comprehensive understanding of safety and efficacy of SBRT for localized PCa, King et al. (2013) recruited 1100 localized PCa patients (58% low-, 30% intermediate-, and 11% high-risk) with a median follow-up of 3 years in a pooled analysis of prospective clinical trial. The median prescription dose was 36.25 Gy/4–5 fractions in SBRT treatment. The results were promising with a 5-year bPFS rate of 93% for all patients. Furthermore, the 5-year bPFS rates for patients with low risk, intermediate risk and high risk disease were 95%, 84% and 81%, respectively (*P* < 0.001). Moreover, the study conducted by Kishan et al. including 12 phase 2 trials analyzed 2142 PCa patients. Among these patients, 1185 (55.3%) had low-risk disease, while 692 (32.3%) had favorable intermediate-risk PCa, 265 (12.4%) unfavorable intermediate-risk PCa. After a median follow-up of 6.9 years, 7-year bPFS rates were 95.5% for low-risk disease, 91.4% for favorable intermediate-risk disease, 85.1% for unfavorable intermediate-risk PCa, and 89.8% for all intermediate-risk PCa. Only 0.60% patients had grade 3 or worse acute GU toxicity, while 0.09% experienced grade 3 or worse acute GI adverse reaction. Additionally, 2.4% and 0.4% patients had grade 3 or worse late GU and GI toxicity, respectively (Kishan et al. 2019). The results were consistent with our observation. In our study, 133 patients (10 low-, 21 favorable intermediate-, 31 unfavorable intermediate-, 45 high-, and 26 very high risk cases) received SBRT. After a median follow-up of 57.7 months, the 5-year bPFS rate was 83.6% for all patients. Additionally, the 5-year bPFS rates for low-, favorable intermediate-, unfavorable intermediate-, high-, and very high risk PCa patients were 87.5%, 95.2%, 90.5%, 86.3% and 61.6%, respectively. Since we have included more high and very high risk PCa patients, the 5-year bPFS rate was slightly lower than that in previous studies. It should be addressed that the 5-year bPFS rate for low risk patients are needed for further evaluations due to the limited patients enrolled. Kishan AU and his co-workers found that the treatment toxicities were mild with 0.8% patients reporting grade 3 acute GU adverse reaction and 0.8% patient experiencing grade 3 late GU toxicity. In our study, the occurrence rate of acute and late toxicities was significantly lower than that in previous studies. This could be caused by the low rate of patients with ADTenrolled in our study (38.3%, 51/133).

There were some limitations in our study. First, due to the retrospective nature and a small sample size, generalization of the results should be cautious. Second, the safety and feasibility of SBRT for PCa patients were the main focus, therefore, further comparisons with conventional fractionated radiotherapy are required. Moreover, the baseline of patients enrolled in our study was heterogeneous with a wide range of ages as well as lesion sizes. What’s more, due to the retrospective nature, the occurrence rate of acute and late toxicities was significantly lower than in previous studies. There were 36 (27.1%) patients present of ADT before SBRT. The reduced irradiation volume may reduce adverse events. Therefore, a longer follow-up in larger prospective cohort studies is warranted to compare patients’ outcomes and adverse effects of surgical resection with those of SBRT and conventional fractionated radiotherapy, which may be beneficial for accurate decision making of treatment options.

## Conclusion

SBRT is a safe and effective treatment with an encouraging bPFS rate and tolerable toxicity for localized PCa patients. Patients with a Gleason score < 8 and relatively low risk disease had a better biochemical control rate. Moreover, patients with a large prostate volume had a similar outcome comparing to those with a small prostate volume. Notably, more prospective clinical trials are needed to evaluate the efficiency and safety of SBRT for patients with localized PCa.

## Data Availability

The datasets generated for this study are available on request to the corresponding author.

## Abbreviations

SBRT: Stereotactic body radiation therapy
PCa: Prostate cancer
bPFS: Biochemical progression-free survival
CTCAE: Common terminology criteria for adverse events
LC: Local control
OS: Overall survival
DPFS: Disease progression-free survival
GU: Genitourinary
IGRT: Image-guided radiotherapy
IMRT: Intensity-modulated radiotherapy
ADT: Androgen deprivation therapy
MRI: Magnetic resonance imaging
ECT: Emission computed tomography
ECOG: Eastern Cooperative Oncology Group
CT: Computed tomography
CTV: Clinical target volume
PTV: Planning target volume
OARs: Organs at risk
PSA: Prostate-specific antigen
GI: Gastrointestinal
